# Stressors, mental health and coping amongst forcibly displaced youth since the advent of COVID-19: A systematic review

**DOI:** 10.1177/25161032251388295

**Published:** 2025-10-09

**Authors:** Maureen Seguin, Robin Cavagnoud, Camila Gianella, Taras Khomych, Natalia Vibla

**Affiliations:** 1 Department of Public Health, Environments and Society, Faculty of Public Health and Policy, 4906 London School of Hygiene and Tropical Medicine, London, UK; 2 Department of Social Sciences, 42692 Pontificia Universidad Católica del Perú, Lima, Peru; 3 Department of Theology, Philosophy and Religious Studies, 4588 Liverpool Hope University, Liverpool, UK; 4 School of Law and Criminology, 4588 Liverpool Hope University, Liverpool, UK

**Keywords:** mental health, youth, refugee, internally displaced person, coping

## Abstract

Mental health is a key issue for forcibly displaced youth. The evidence base on the mental health of youth forcibly displaced since the start of the pandemic is undefined, as well as sources of stressors and coping approaches. This systematic review aims to identify literature on the mental health of forcibly displaced youth in low- and middle-income settings, with focus on displacement since the advent of the COVID-19 pandemic. Objectives are to examine (1) sources of stress, (2) prevalence and covariates of common mental disorders (CMDs) and (3) coping approaches. Six databases were searched in February 2023. Search terms focused on CMDs, stress and forcibly displaced populations. Articles based on data collected after the onset of the COVID-19 pandemic focused on forcibly displaced persons aged 10-29 were included. Quantitative observation and intervention studies reporting CMD prevalences and related concepts were included, as were qualitative studies about stressors and/or coping approaches. Prevalences of CMDs and covariates were tabulated. Inductive thematic coding was conducted on qualitative data on stressors and coping. Interpretation of coping data was guided by a taxonomy including problem solving, support seeking, distraction/avoidance and positive cognitive restructuring. Twenty-one articles were included. Economic issues were the most prominent source of stress and led to subsequent stressors. Depression and anxiety symptom prevalence ranged from 6.2% to 77.4% and 17.2%–32.8% respectively. Problem-solving and support seeking were the most common coping approaches. Supporting the mental health and coping approaches of this marginalised group is critical to recovery in the post-COVID era.

## Background

At the end of 2022 there were approximately 108.4 million forcibly displaced persons worldwide, including 34.6 million refugees and 57.3 million internally displaced persons (IDPs) (UNHCR, 2023). An estimated 43.3 million are aged 17 and younger (UNICEF, 2023). Mental health is recognised as a key public health issue for the forcibly displaced (Tol et al., 2023), especially for children and adolescents as displacement-related trauma risks their physical, emotional and social development (Ramaiya et al., 2023; Reed et al., 2012).

At the onset of the COVID-19 pandemic, global clinical rates of anxiety and depression amongst youth stood at 11.6% and 12.9% respectively (Lu, 2019; Tiirikainen et al., 2019). Comparable global rates during the pandemic increased to 25.2% and 21.0% for depression and anxiety (Racine et al., 2021), indicating an increase in poor mental health correlated with the pandemic. Depressive symptoms ranged from 4% to 75%, and anxiety from 5% to 54% in a recent review on pandemic impacts on adolescents in low- and middle-income countries (LMICs) (Ramaiya et al., 2023). Within LMICs, the worst mental health outcomes were observed for the most marginalised youth, including refugees. This may reflect the unique stressors such youth may face, such as security threats, political instability, poor infrastructure, and fragile health systems (Pedersen, 2002; Reed et al., 2012), and justifies the need to review mental health outcomes of forcibly displaced adolescents and young people in LMICs since the start of the pandemic.

It is important to recognise that not all forcibly displaced youth develop mental illness. According to the risk and resilience model (Suárez-Orozco et al., 2018), psychological outcomes are shaped by (1) individual characteristics (i.e. developmental competencies, ethnicity and gender), (2) ‘microsystems’ (i.e. family, school and peers) (3) contexts of reception (i.e. resettlement programs, societal attitudes, educational resources, and family dynamics), and (4) global forces (i.e. war and climate change). Though forcibly displaced adolescents are impacted by these factors, they also demonstrate agency in their interactions within these four contextual levels (Abdi et al., 2023).

A range of positive coping mechanisms amongst adolescents in LMICs have been observed, including volunteering, seeking social support, and building resilience (Ramaiya et al., 2023). A review of coping strategies amongst forcibly displaced adults in LMICs found that seeking support and taking a problem-solving approach were protective against poor mental health, whilst avoidance techniques (such as substance use and/or ignoring problems) were linked to poorer outcomes (Seguin & Roberts, 2017). To date, coping approaches used by forcibly displaced youth in LMICs has not been comprehensively reviewed since the start of the pandemic. This review builds on these findings by compiling published literature on mental health outcomes, risk and protective factors amongst forcibly displaced adolescents and young adults in LMICs, with specific focus on data collected since the onset of the COVID-19 pandemic.

### Definition of the research problem

Despite recognition of mental health as a priority issue for forcibly displaced youth, key knowledge gaps are evident regarding the prevalences of CMDs for this group, their stressors, and coping approaches. Defining the evidence base in these areas is critical to developing interventions to best target stressors, support coping approaches, and enhance mental health for this vulnerable group in LMICs. Thus, findings may be useful for policymakers, mental health professionals, and other stakeholders involved in mental health service development and provision for forcibly displaced youth in LMICs.

## Objectives

Our objectives are to examine: (1) sources of stress, (2) prevalence and covariates of common mental disorders (CMDs), and (3) coping approaches used to deal with stressors. It is important to address these objectives within the context of COVID-19 due to the evident increase in CMD prevalence amongst youth during the pandemic (Racine et al., 2021), especially amongst displaced youth in LMICs (Ramaiya et al., 2023). As the broad impacts of the pandemic on children and young people will be among most lasting societal consequences (United Nations, 2020), addressing these objectives is critical to develop appropriate interventions supporting mental health and coping approaches for this marginalised group.

## Study selection and analysis

### Search strategy

Our approach follows the Preferred Reporting Items for Systematic Review and Meta-Analyses guidelines (Moher et al., 2009). Six databases (Medline, PsychINFO, Global Health, EMBASE, Web of Science and LILACs) were searched in February 2023. Search terms focused on CMDs (i.e. common mental disorder* or mental health or mental disabil* or mental* or psych* or anxiety disorder* or depress* or post-traumatic stress disorder* or posttraumatic stress disorder* or PTSD or somat* or alcohol* or drug), stress (stress* or challeng* or worr* or difficult* or strain* or struggle*) and forcibly displaced populations (internally displace* or displaced persons or forced migra* or refugee* or migra* or genocide* or armed conflict* or conflict-affect* or war-affect* or war). Where available, MeSH terms were also integrated into the search strategy. Translated search terms were utilised in a November 2023 SciELO search to access South American articles. Database searches were complemented by manual searches of reference lists of included articles and forward-citation Google scholar searches. Grey literature was identified through searches of non-governmental organisation (NGO) websites focused on displaced children, adolescents, and young adults.

### Eligibility criteria

Articles and book chapters set in low-income, lower-middle income and upper-middle countries containing data on mental health, stressors or coping amongst forcibly displaced youth were included. ‘Forced displacement’ refers to situations where individuals and/or communities have been compelled to leave their places of origin due to war, violence, human rights abuses, and/or natural or man-made disasters (UNHCR, 2010). We included refugees, asylum seekers, IDPs, and forced migrants in our conception of forcibly displaced youth.

Refugees are defined as persons who are unable or unwilling to return to their country of origin because of fear of persecution due to race, religion, nationality, membership of a particular social group, or political opinion (UNHCR, 2025a). Like refugees, those seeking asylum relocate in response to unsafe conditions in their countries of origin. They may eventually attain legal refugee status, but not in advance of arrival (Abdi et al., 2023). In the United Kingdom and other countries, asylum seekers may face long wait times while their application for asylum is considered. Those who make a successful claim are then granted refugee status. Internally displaced persons may leave their areas of origin for the same reasons as refugees and asylum seekers, but do not cross international borders (UNHCR, 2025b). The term ‘migrant’ is interpreted in many different ways, and is often conflated with immigration status, race and ethnicity (Anderson & Blinder, 2024). As we are interested in data on forcibly displaced samples, we included papers on migrants they appeared to have relocated for reasons similar to refugees, asylum seekers and IDPs.

We included qualitative and quantitative articles published in English, French and Spanish, reflective of the language proficiencies of coauthors. Of quantitative articles, we included observation and intervention studies reporting the prevalence of CMDs (including anxiety, depression, somatoform, and substance-related disorders) and related concepts such as psychosocial and subjective wellbeing, and/or coping and/or resilience measures. Qualitative studies about perceived stressors and/or coping approaches were included.

Articles based solely on data collected before the start of the pandemic were excluded, as well as data within longitudinal studies collected before the pandemic. Case studies, editorials and Masters and PhD theses were excluded. We excluded articles focused solely focused on war veterans and combatants, as their sources of stress and coping strategies may also differ significantly from forcibly displaced civilians. Articles on youth who had migrated, but were not forced to move (i.e., international students) were excluded.

We focused on persons aged from 10–29. Samples which contained ages younger than 10 or older than 29 were included if the mean age fell within 10–29. We drew on the World Health Organisation definition of adolescence (10–19) (WHO, 2023) and a definition of emerging adulthood (Hochberg & Konner, 2020) in setting this age range. For the remainder of the review, we will refer to the samples as ‘youth.’ We defined coping as cognitive and behavioural efforts made to master, tolerate, or reduce internal and external demands (Folkman & Lazarus, 1980).

### Selection process

Results of the searches above were downloaded into Endnote. The first author screened titles and abstracts against the inclusion and exclusion criteria and accessed full texts of articles meeting these criteria. Articles fulfilling inclusion criteria were included.

### Data extraction and analytic approach

Author(s), year of publication, article type, data collection dates, origin and host country, displacement type (refugees, internally displaced and/or migrant), age range, gender breakdown, sample size, methodological approach, and objective addressed were extracted.

Heterogeneous research designs and assessment tools for mental health conditions and coping approaches precluded a pooling of results extracted from quantitative articles. Therefore, a narrative description of quantitative results is provided rather than a metanalysis. Inductive thematic coding was conducted on qualitative data on stressors and coping. The analysis of coping strategies was guided by a 5-part taxonomy including problem solving (instrumental action), support seeking (reaching out to others to receive support), distraction/avoidance (engaging in pleasurable activities), and positive cognitive restructuring (changing one’s perspective of a stressor to see it more positively) (Skinner et al., 2003).

### Quality assessment

The quality of quantitative, qualitative and mixed methods articles was assessed by the Strengthening the Reporting of Observational studies in Epidemiology checklists (von Elm et al., 2008), the Critical Appraisal Skills Program checklist (Critical Appraisal Skills Programme, 2018) and the Mixed Methods Appraisal Tool (Hong et al., 2019) respectively. Articles were not excluded based on quality. Rather, these assessments were conducted to examine the quality of the evidence base.

## Findings

### Study selection

**Figure 1.**
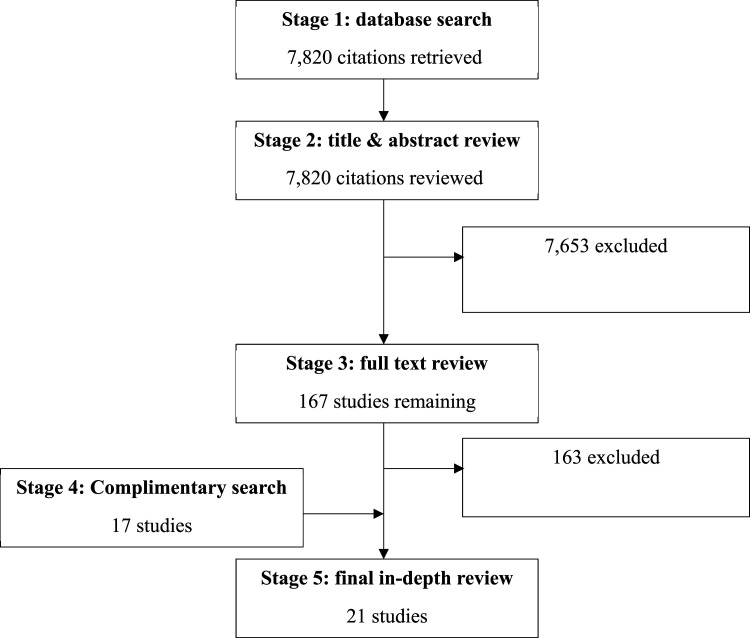
Screening results.

The search results are summarised in Figure 1 below. A total of 10,806 records were identified in the database search, from which 2,986 duplicates were removed, leaving 7,820 for title and abstract screening. Stage 2 excluded 7,653 entries based on exclusion criteria and duplication. Full text review was conducted on 167 articles. This is a relatively high number due to the need to manually check articles for data collection dates and age ranges. It also reflects the nuanced nature of the concept of coping, and the need to access full texts to judge whether authors’ usage of this concept aligned with the coping conceptualisation used in this paper (Folkman & Lazarus, 1980). Four articles from the main database search met inclusion criteria. Articles were mainly excluded due to data collection occurring prior to the pandemic, or exclusive focus on children aged 9 or younger. Complementary searching, including manual searches of reference lists of included articles and forward-citation Google scholar searches produced 17 studies, yielding a total of 21 articles.

Details for the 21 articles are shown in Table 1. Included are 13 journal articles and 1 book chapter. Six citations are grey literature, comprised of 5 research reports and 1 policy brief. Sample sizes for quantitative and mixed methods articles range from 115 to 2801, and qualitative from 10 to 224. Of studies that reported gender, the majority reported approximately half-and-half splits, with one recruiting exclusively females (Baird et al., 2022) and one exclusively males (Mohammadsadeghi et al., 2022).

**Table 1.** Details of included studies.

Author(s) (Year)	Article type^a^	Data collection: Mm/yy-mm/yy	Origin/host country (displacement type)^b^	Sample size (gender%, age range, age mean)	Methodology^c^	Objective addressed^d^	Quality (H, M. L)
Abu Hamad et al. (2022)	JA	10/20-01-21	Palestine/Palestine (IDP)	QT = 147, QL = 61 (♀♂?, 12–19)	MM (CSS, SSI)^e^	2, 3	M
Abu Hamad, Jones, and Pincock (2021)	BC	?	Unclear/Palestine, Ethiopia, Jordan, Lebanon and Bangladesh (IDP & R)	224 (♀♂?, 10–19)	QL (SSI, FGD, P, AD).	1, 3	H
Abu Hamad, Baird, et al. (2021)	GL(R)	10/20-12/20	Syria & Palestine/Jordan (R)	1201 (♀♂?, 11-19	MM (SSI, CSS)	1, 2, 3	H
Baird et al. (2022)	JA	04/20-07/20	Myanmar & Syria/Bangladesh & Jordan (R)	667 (667♀, 16–18)	MM (CSS, SSI)^e^	1, 2, 3	H
Devonald et al. (2022)	JA	06/21-07/21	Palestine & Syria/Lebanon (R)	61 (♀♂?, 16–25)	QL (FGD, PO, CM)	1	H
Gómez-Restrepo et al. (2023)	JA	10/21-03/22	Colombia/Colombia (IDP)	657 (58♀, 42♂, 12-18, $\bar{x}$ = 14)	QT (CSS)	1, 2, 3	H
Guglielmi et al. (2020a)	JA	05/20-06/20	Myanmar/Bangladesh (R)	QT = 692, QL = 21 (♀♂?, 11–20)	MM (CSS & SSI)	1, 2, 3	H
Guglielmi et al. (2020b)	GL(B)	05/20-06/20	Myanmar/Bangladesh (R)	QT = 725, QL = 21 (♀♂?, 10–19)	MM (CSS, SSI)^f^	1, 3	M
Jamaluddine and Sieverding (2022)	GL(R)	08/20-10/20, 02/21-03/21	Syria/Jordan (R)	1757 (52♀, 48♂, 16–30)	QT (CSS)	2	H
Jones et al. (2021)	JA	07-12/2020	Ethiopia & Syria/Ethiopia & Jordan (M & R)	>32 (>16♀, >16♂, 15–19)	QL (RI)	1, 3	
Jones et al. (2022)	JA	05/20-07/20, 11/20-01/21	Palestine & Syria/Jordan (R)	QT = 2801, QL = ? (50♀, 50♂, 10–21)	MM (LS, SSI)	1, 2, 3	H
Logie et al. (2021)	JA	02/20	South Sudan & Democratic Republic of the Congo/Uganda (R)	58 (50♀, 50♂, 16-24, $\bar{x}$ = 20.9)	QL (SSI, FGD)	1, 3	M
Logie, Okumu, Loutet, Berry, Taing, et al. (2022)	JA	03/21	South Sudan/Uganda (R)	115 (51♀, 49♂, 16-24, $\bar{x}$ = 19.7)	QT (CSS)	2	M
Logie, Okumu, Loutet, Berry, Lukone, et al. (2022)	JA	12/20-02/21	South Sudan/Uganda (R)	120 (50♀, 50♂, 16-24, $\bar{x}$ = 19.7)	QT (INT)	2, 3	H
Mitu et al. (2022)	JA	??/21	Myanmar/Bangladesh (R)	57 (49♀, 51♂, 15–19)	QL (SSI, FGD)	1, 3	M
Mohammadsadeghi et al. (2022)	JA	??/21	Afghanistan/Iran (R & M)	10 (100♂, 19-45, $\bar{x}$ = 26)	QL (SSI)	1, 3	H
Plan International (2021)	GL(R)	12/20	Syria/Lebanon (R)	202 (♀♂?, 10–17)	QT (CSS)	1, 3	L
Presler-Marshall et al. (2021)	GL(R)	??/20	Palestine/Jordan, Lebanon and Palestine (R)	QT = 1248, QL=>50 (♀♂?, 10-12 & 15–17)	MM (CSS, SSI, PR)	1, 3	H
Presler-Marshall et al. (2022)	GL(R)	12/20-07/21	Palestine & Syria/Lebanon (R)	>25 (40♀, 60♂, 15–24)	QL (SSI, FGD)	1, 3	H
Sakyi and Johnson (2022)	JA	07/21-08/21	17 African countries & Pakistan/Ghana (R)	399 (♀♂?, 15–29)	QT (CSS)	2	H
Youssef et al. (2022)	GL(R)	??/19-??/22	Syria/Lebanon (R)	30 (67♀, 33♂, 15–21)	QL (RI, FGD)	1, 3	H

^a^BC = Book chapter, GL(B) = Grey literature (brief), GL(R) = Grey literature (report), JA = Journal article.

^b^IDP = Internally displaced person, M = Migrant, R = Refugee.

^c^AD = Audio diaries, CM-Community mapping exercise, CSS = Cross-sectional survey, INT = Intervention study, LS = Longitudinal surveys, MM = Mixed Methods, P = Photography, PO = Participant observation, PR = Participatory research, QL = Qualitative, QT = Quantitative, RI = Repeat interviews SSI = Semi-structured interviews.

^d^1 = Sources of stress, 2 = Prevalence of common mental disorders, 3 = Coping approaches.

^e^Only QT data extracted as QL data not disaggregated for displaced participants.

^f^Only QL data extracted as QT data not disaggregated for displaced participants.

There was diversity in methodological approaches, with 8 qualitative, 7 mixed methods, and 6 quantitative articles. Data collection occurred from February 2020 to March 2022, with the bulk occurring in the second and fourth quarters of 2020 and the first quarter of 2021.

Within our target age range of 10–29, the years 15–21 were included most often, with high coverage of ages 16-17. There were comparatively fewer articles focused on those aged 10–14 and 22 or older. Almost half of the studies included displaced youth from Syria, followed by frequency by those from Palestine and Rohingyas from Myanmar. A cluster of 3 studies focused on persons from South Sudan, and one each from Colombia, the Democratic Republic of Congo, Afghanistan and Ethiopia. One study focused on a sample of refugees in a Ghanan refugee camp, including youth from 17 African countries and Pakistan (Sakyi & Johnson, 2022).

### Quality assessment

Quality assessment outcomes are reported in Table 1. Common weaknesses were a lack of justification for mixed methods (for mixed methods articles), a lack of consideration of the relationship between the researcher(s) and participants (for qualitative articles), and a lack of discussion regarding sources of bias (for quantitative articles).

### Sources of stress

Fifteen articles focused on sources of stress. Findings for quantitative articles are shown in Table 2, organised by the following prominent themes: economic, hunger/health, education and early marriage.

**Table 2.** Sources of stress.

Stressor	Percentage (%) experiencing stressor^a^	Increase versus before COVID (*p* value)
Economic	78.2/63.2^b^ family member lose job (Syrian) (Baird et al., 2022).	*p* < .001 lost job (Syrian, HC^c^) (Jones et al. (2022).
	73 basic needs unmet (Plan International, 2021).	*p* < .01 lost job (Syrian, ITS^c^) (Jones et al., 2022)
	72.5/80.4^b^ unable to buy food (Rohingya) (Baird et al., 2022).	*p* < .05 lost job (Syrian, C^c^) (Jones et al., 2022).
	69 (girls) unable to buy menstrual pads (Plan International, 2021).	*p* < .001 lost job (Palestinian) (Jones et al. (2022).
	66.7/52.5^b^ family member lose job (Rohingya) (Baird et al., 2022).	*p* < .01 lost income (Syrian, HC^c^) (Jones et al., 2022).
	65 unable to buy masks (Plan International, 2021).	*p* < .01 lost income (Palestinian) (Jones et al., 2022).
	50.0/43.2^b^ unable to buy food (Syrian) (Baird et al., 2022).	*p* < .001 unable to buy food (Syrian, HC^c^) (Jones et al., 2022).
	41 unable to buy non-food items (ITS, C^c^) (Abu Hamad, Baird, et al., 2021).	*p* < .05 unable to buy food (Palestinian) (Jones et al., 2022).
	35 pressure to work (Palestinian ♂ in Jordan) (Presler-Marshall et al., 2021).	
	30 face economic challenges (Plan International, 2021).	
	23 unable to buy menstrual pads (Abu Hamad, Baird, et al., 2021).	
	16 unable to buy medications (Abu Hamad, Baird, et al., 2021).	
	13 unable to pay bills (Plan International, 2021).	
Hunger/Health	51 feared COVID (Plan International, 2021).	*p* < .001 food insecure (Syrian, HC^c^) (Jones et al., 2022).
	28.3/24.3^b^ hunger (Rohingya) (Baird et al., 2022).	*p* < .001 food insecure (Syrian, ITS^c^) (Jones et al., 2022).
	27 hunger (Abu Hamad, Baird, et al., 2021).	*p* < .001 hunger in past week (Syrian, HC^c^) (Jones et al., 2022).
	22 hunger (girls) (Guglielmi et al., 2020a).	
	22 lack of food (Plan International, 2021).	
	14 hunger (boys) (Guglielmi et al., 2020a).	
	13.7/14.9^b^ hunger (Syrian) (Baird et al., 2022).	
	12.8/16.8^b^ worse self-reported health (Syrian) (Baird et al., 2022).	
	11.3/4.9^b^ worse self-reported health (Rohingya) (Baird et al., 2022).	
	8.5 worse self-reported health (♀7/♂12) (Guglielmi et al., 2020a).	
Education	47, 32 connectivity problems (C, ITS^c^) (Abu Haham et al., 2021b).	N/A
	46 enrolled (ITS^c^) (Abu Hamad, Baird, et al., 2021).	
	45 dissatisfied with distance learning (Plan International, 2021).	
	44 unable to attend school (Plan International, 2021).	
	35 enrolled in school (boys, aged 16–20) (Guglielmi et al., 2020a).	
	6 enrolled in school (girls, aged 16–20) (Guglielmi et al., 2020a).	
Early marriage	18, 12 (ITS, C^c^) (Abu Hamad, Baird, et al., 2021).	*p* < .001 (Syrian, ITS) (Jones et al., 2022).
		*p* < .01 (Palestinian) (Jones et al., 2022).
		*p* < .05 (Syrian, host community) (Jones et al., 2022).

^a^Arranged in descending order in percentage by stressor category.

^b^Figures refer to ‘ever married’ and ‘never married’ respectively.

^c^C = Camp, HC = Host Community, ITS = Informal Tented Settlement.

Economic challenges resulted from family members losing jobs (Baird et al., 2022; Plan International, 2021), leading to poverty and food and housing insecurity (Abu Hamad et al., 2021a, 2021b; Baird et al., 2022; Devonald et al., 2022; Guglielmi et al., 2020a, 2020b; Jones et al., 2021, 2022; Logie et al., 2021; Mitu et al., 2022; Mohammadsadeghi et al., 2022; Plan International, 2021; Youssef et al., 2022). Decreased job availability was sometimes paralleled by decreased social protection initiatives, compounding the impact (Abu Hamad, Jones, & Pincock, 2021). Youth felt pressure to take up work (Presler-Marshall et al., 2021), including risky jobs in illicit industries (Abu Hamad, Jones, & Pincock, 2021; Youssef et al., 2022). Whilst some youth felt the lack of outside employment contributed to familial violence (Abu Hamad, Jones, & Pincock, 2021; Logie et al., 2021; Presler-Marshall et al., 2021), others thought spending time together benefitted family dynamics (Presler-Marshall et al., 2021).

Those living in refugee camps reported overcrowded conditions, limited access to resources, and a lack of hygiene and social distancing measures (Abu Hamad, Jones, & Pincock, 2021; Devonald et al., 2022; Jones et al., 2021; Logie et al., 2021; Mitu et al., 2022; Youssef et al., 2022). Further housing issues included a lack of privacy, insulation, heating, ventilation, sewage draining, waste removal, water, electricity, and cooking gas (Devonald et al., 2022; Mitu et al., 2022; Youssef et al., 2022).

Youth who managed to remain enrolled in education had difficulty accessing online learning due to limited access to devices, internet and electricity (Abu Hamad et al., 2021a, 2021b). Low levels of enrollment were reported, ranging from 6% (Guglielmi et al., 2020a) to 46% (Abu Hamad, Baird, et al., 2021), indicating widespread inaccessibility.

Of articles examining early marriage, most identified this as a stressor (Abu Hamad, Baird, et al., 2021; Presler-Marshall et al., 2021; Youssef et al., 2022). Conversely, some youth felt less pressure to marry than before the pandemic, attributed to the lack of economic capital required to hold a wedding, start a family and establish a household (Abu Hamad, Baird, et al., 2021; Jones et al., 2022).

### Gender differences regarding sources of stress

Qualitative articles outlined large differences between boys’ and girls’ experiences of being housebound due to lockdowns. Though several studies (Abu Hamad, Jones, & Pincock, 2021; Guglielmi et al., 2020a; Jones et al., 2021; Youssef et al., 2022) noted that girls already had less freedom to socialise and travel outside the home prior to the pandemic, lockdowns resulted in further movement restrictions (Abu Hamad, Jones, & Pincock, 2021; Jones et al., 2021), for instance, seeing maternal relatives (Mitu et al., 2022). Limited access to mobile phones, high transportation costs and financial difficulties created barriers for married girls to access support networks (Youssef et al., 2022) and information about COVID (Guglielmi et al., 2020a). Girls with disabilities were particularly isolated during lockdowns (Abu Hamad, Jones, & Pincock, 2021). Lockdowns increased the share of household chores and child-rearing responsibilities for girls, to which their male counterparts were not expected to contribute (Abu Hamad, Jones, & Pincock, 2021). Because girls were more housebound than boys, poor housing quality including lack of ventilation, water and privacy disproportionately impacted girls (Mitu et al., 2022; Youssef et al., 2022).

Gender-based differences were also apparent regarding educational opportunity. Inability to pay school fees pushed boys into work (Abu Hamad et al., 2021a, 2021b; Youssef et al., 2022). Girls who were engaged or married had especially restricted education access (Presler-Marshall et al., 2022).

### Prevalence and covariates of common mental disorders

As shown in Table 3, 8 quantitative articles reported prevalence of common mental disorders (Abu Hamad et al., 2021b, 2022; Gómez-Restrepo et al., 2023; Guglielmi et al., 2020a; Jones et al., 2022; Logie et al., 2022a, 2022b; Sakyi & Johnson, 2022) and two of psychosocial and subjective wellbeing (Baird et al., 2022; Jamaluddine & Sieverding, 2022).

**Table 3.** Prevalence of Common Mental Disorders, descending order by symptom prevalence.

Author(s) and (year)	Symptoms (%)	Diagnosis (%)	Measurement tool
Depression			
Logie, Okumu, Loutet, Berry, Lukone, et al. (2022)	77.4		PHQ-2
Gómez-Restrepo et al. (2023)	30		PHQ-8
Logie, Okumu, Loutet, Berry, Taing, et al. (2022)	28.7		PHQ-9
Jones et al. (2022)	16.0–13.0^a^, 13.0–8.0^b^,12.0–7.0^c^, 14.0–10.0^d^		PHQ-8
Abu Hamad et al. (2022)		8.3	PHQ-8
Abu Hamad, Baird, et al. (2021)	8.0, 8.0^e^		PHQ-8
Guglielmi et al. (2020a)	6.2		PHQ-8
Anxiety			
Sakyi and Johnson (2022)	21.1, 32.8^f^		CAS
Gómez-Restrepo et al. (2023)	18.9		GAD-7
Abu Hamad et al. (2022)		17.2	GAD-7
Abu Hamad, Baird, et al. (2021)	10.0. 7.0^g^		
PTSD			
Gómez-Restrepo et al. (2023)	22.3		PCL-5
Substance use			
Gómez-Restrepo et al. (2023)	17.2		SRQ
Psychosocial and subjective wellbeing			
Baird et al. (2022)	90.4, 89.3 worried/anxious, 88.5, 85.4 scared/fearful (Rohingya)		Bespoke tool
	68.4, 58.4 worried/anxious, 69.2, 60.2 scared/fearful (Syrian)^h^		
Jamaluddine and Sieverding (2022)	52.0 experienced poor subjective wellbeing		WHO-5 wellbeing index

^a^For Syrians living in host communities, at times 1 and 2.

^b^For Syrians living in informal tented settlements, at times 1 and 2.

^c^For Syrians living in camps, at times 1 and 2.

^d^For Palestinians, at times 1 and 2.

^e^For Palestinians and Syrians living in informal tented settlements and camps respectively.

^f^For ages 15-19 and 20-29 respectively.

^g^For Palestinian and Syrian living in camps and informal tented settlements respectively.

^h^For ever married and never married respectively.

Psychosocial and subjective wellbeing were examined in two articles (Baird et al., 2022; Jamaluddine & Sieverding, 2022). High levels of worry and anxiety were observed amongst 90.4% (married) and 89.3% (never married) Rohingya teenagers, and 68.4% (married) and 58.4% (never married) Syrian refugees (Baird et al., 2022). Over half of a sample of Syrian refugees in Jordan experienced poor subjective wellbeing, predicted by being aged 18–24 (versus 16-17), not attending school, being unemployed, being married or divorced (versus never married), receiving only 1-2 cash transfers (rather than 3), and living in an urban or camp location (rather than rural) (Jamaluddine & Sieverding, 2022).

Of all CMDs, depression prevalence was the most reported, featuring in 7 articles (Abu Hamad et al., 2021b, 2022; Gómez-Restrepo et al., 2023; Guglielmi et al., 2020a; Jones et al., 2022; Logie et al., 2022a, 2022b). Symptom prevalence ranged from a low of 6.2% amongst Rohingya refugees in Bangladesh (Guglielmi et al., 2020a) to a high of 77.4% amongst South Sudanese refugees in Uganda (Logie, Okumu, Loutet, Berry, Lukone, et al., 2022). Predictors included family dysfunction (Gómez-Restrepo et al., 2023) and water insecurity (Logie, Okumu, Loutet, Berry, Taing, et al., 2022). Female gender was significantly associated with depression amongst young Colombian IDPs (Gómez-Restrepo et al., 2023), but non-significant amongst South Sudanese youth in Uganda (Logie, Okumu, Loutet, Berry, Lukone, et al., 2022).

Three studies measured anxiety. Prevalences ranged from a high of 32.8% (for those aged 20–29) and 21.1% (for those aged 15–19) (Sakyi & Johnson, 2022), followed by 18.9% amongst Colombian IDPs (Gómez-Restrepo et al., 2023) and 17.2% amongst Palestinian IDPs (Abu Hamad et al., 2022). Significant associations were found between anxiety and female gender (Gómez-Restrepo et al., 2023; Sakyi & Johnson, 2022) and older age (15–18 (Gómez-Restrepo et al., 2023) and 20–29 (Sakyi & Johnson, 2022)) versus younger counterparts. Only one study (Gómez-Restrepo et al., 2023) measured PTSD and substance abuse, reporting prevalences of 22.3% and 17.2% respectively amongst IDPs in Colombia. PTSD was significantly associated with female gender and severe family dysfunction.

### Coping approaches

Seventeen articles focused on coping (Abu Hamad et al., 2021a, 2021b, 2022; Baird et al., 2022; Gómez-Restrepo et al., 2023; Guglielmi et al., 2020a, 2020b; Jones et al., 2021, 2022; Logie et al., 2021, 2022b; Mitu et al., 2022; Mohammadsadeghi et al., 2022; Plan International, 2021; Presler-Marshall et al., 2021, 2022; Youssef et al., 2022). There was a prominent focus on coping specifically with the financial insecurity created and compounded by COVID-19.

A variety of problem-solving tactics were reported in qualitative and quantitative articles, including quitting education to work, selling possessions, and reducing expenditure for food and other necessities (Abu Hamad, Baird, et al., 2021; Jones et al., 2021; Plan International, 2021; Presler-Marshall et al., 2022; Youssef et al., 2022). Some engaged in illicit or illegal activities (Youssef et al., 2022), including drug dealing (Mitu et al., 2022), begging (Jones et al., 2022), working despite lockdowns (Jones et al., 2021), and/or engaging in transactional sex or early marriage (Logie et al., 2021). Some attended vocational training and cash-for-work programmes, which were more accessible to boys than girls (Presler-Marshall et al., 2022; Youssef et al., 2022). Some coped with financial instability through planned or attempted migration (Jones et al., 2022; Presler-Marshall et al., 2022; Youssef et al., 2022), in some cases being trafficked for marriage (for girls) or work (for boys) (Mitu et al., 2022; Presler-Marshall et al., 2021). To cope with rising housing costs, samples reported moving to homes of extended family and/or cheaper housing and using firewood for heating and cooking (Mitu et al., 2022; Youssef et al., 2022). Coping approaches to rising costs associated with health care included using traditional practices (such as homebirths and home remedies) and seeking health information online (Abu Hamad, Jones, & Pincock, 2021; Youssef et al., 2022).

Financial, emotional and material support was sought from family, friends, and charities (Abu Hamad et al., 2021a, 2021b; Jones et al., 2021, 2022; Mohammadsadeghi et al., 2022; Plan International, 2021; Presler-Marshall et al., 2021, 2022; Youssef et al., 2022). Reaching out to friends through social media was frequently mentioned (Jones et al., 2022), which was more accessible to boys than girls (Abu Hamad, Jones, & Pincock, 2021; Youssef et al., 2022). Some youth engaged in cognitive restructuring by seeking guidance from religion (Guglielmi et al., 2020a, 2020b; Jones et al., 2022; Youssef et al., 2022). Approximately 90% and 60% of Rohingya and Syrian refugees respectively thought things would be better in a year (Baird et al., 2022), indicating a hopeful attitude for the future.

Displaced youth engaged in distraction and avoidance techniques including reading, writing, playing games with siblings, and doing chores (Jones et al., 2022). Negative approaches were also reported, such as excessive sleeping, self harm and attempted and suicidal activity (Youssef et al., 2022). Findings on smoking were equivocal. Though an increase in smoking was observed in one study (Youssef et al., 2022), others found evidence of reduced smoking less due to lack of money to buy cigarettes (Abu Hamad et al., 2021a, 2021b; Jones et al., 2022). Gender differences were observed regarding distraction techniques, due to boys’ better access to mobile phones and outside space (Jones et al., 2021; Mitu et al., 2022; Youssef et al., 2022). Boys coped by socialising with peers outside through games, sport or long walks, and used mobile phones to play online video games. Girls were not permitted to engage in such activities, and instead engaged in domestic work and childcare (Youssef et al., 2022).

## Conclusions and clinical implications

This paper reports the prevalence of and risk factors for CMDs amongst forcibly displaced youth in LMICs, along with key stressors and coping approaches for challenging circumstances since the start of the COVID-19 pandemic.

The centrality of the economic fallout from COVID-19 and containment measures was a salient finding. This prominent stressor led to housing- and health-related stressors and prompted coping responses. Whilst economic issues have long been acknowledged as causing stress for forcibly displaced groups, including in the pre-pandemic literature (Cavagnoud, 2023; Ott & Montgomery, 2015), the magnitude of economic hardship resulting from the pandemic is unique (Espinel et al., 2020; Martuscelli, 2021). Youth in the review perceived a worsening of economic prospects due to COVID-19 compared to previous hardship. Moreover, as noted elsewhere (Hadian et al., 2022), stress and poor mental health resulting from COVID-19 may present barriers to work, thereby weakening sectors striving to recover from the negative economic consequences of the pandemic.

Though COVID-19 impacted economies around the globe, the rate of recovery has been uneven and inequalities between states have widened, particularly impacting forcibly displaced populations (Bojorquez et al., 2021). By 2021, 40% of advanced economies had recovered compared to 27% of middle-income countries and 21% of low-income countries (The World Bank, 2022). Given the link between economic hardship and poor mental health, the slow rate of recovery in LMICs implies that poverty and worsened mental health resulting from the pandemic may linger in these settings. As much of the data in this review was collected in 2020 and 2021, the longer-term mental health consequences are unknown.

Due to the sparse evidence base, particularly for PTSD and substance abuse, we cannot confidently judge the prevalence or covariates of CMDs among forcibly displaced youth in LMICs in the wake of COVID. Depression prevalence ranged from 6.2% to 77.4% and anxiety from 17.2% to 32.8%., which roughly coincide with prevalences reported in a review on the impacts of the pandemic on adolescents (including those not forcibly displaced) in LMICs (Ramaiya et al., 2023). The wide ranges of prevalence for depression and anxiety reflect the scope of assessment tools, diversity in forcibly displaced groups sampled, their experiences, and resources supporting coping approaches.

Age and gender were the most investigated covariates of CMDs. Older age groups had elevated anxiety and worse subjective wellbeing relative to younger counterparts. Most studies found relationships between female gender and worse depression, anxiety and PTSD outcomes (Gómez-Restrepo et al., 2023; Sakyi & Johnson, 2022). This is consistent with findings for adolescents in LMICs (Ramaiya et al., 2023), global pre-COVID findings (Steel et al., 2014) and within forcibly displaced adult populations (Porter & Haslam, 2005; Spiritus-Beerden et al., 2021), underscoring the need to disaggregate mental health findings by gender and justifying future research to examine mechanisms shaping gender-specific mental health outcomes.

Problem-solving and seeking support were the two most prominent coping approaches reported, resonating with pre-COVID reviews amongst adult forcibly displaced populations in LMICs (Peevey et al., 2022; Seguin & Roberts, 2017). Differing coping approaches used by female versus male youth in this review point to the importance of social context in shaping coping responses.

This review reveals several evidence gaps. Though Venezuela, Somalia and Central African Republic ranked within the top 10 origin countries of refugees worldwide in 2022 (UNHCR, 2023), we located no articles focused on youth from these countries. As only two studies focus on youth *migrants* (with the bulk focusing on refugees or IDPs), the stressors, mental health outcomes and coping approaches for youth transiting via irregular migration is largely unknown. Third, we found relatively few studies about youth aged 21 and older.

This review includes not only journal articles but a proportionately large number of grey literature reports. Because screening and data extraction was completed by only one author, potential for selection and extraction bias is present. Due to the context-specific nature of included articles, results may not be generalizable to other settings.

The findings from this review support future research in three areas. First, there is a need for specific focus on older age groups within the larger ‘youth’ bracket. This review observed that older age groups (within the ‘youth’ group) had worse anxiety and subjective wellbeing than younger groups, as noted elsewhere (Ramaiya et al., 2023). Older youth may face different stressors than their younger counterparts, including becoming parents and taking on caregiving roles. Caregiving roles may introduce new social and emotional burdens within the wake of COVID-19 (Sheikhbardsiri et al., 2022), which justifies research attention. Thus, future inquiry focused on older adolescents is merited. Moreover, it indicates the need to disaggregate ‘adult’ age groups (18 and older) often utilised in studies on mental health amongst forcibly displaced populations into smaller age subgroups to view findings for young adults.

Second, the evidence base on coping would be enhanced through greater research attention on the social context of coping approaches. This review observed differences between male and female youth regarding coping approaches, but there is little explanation within included papers on why such differences persist. Expanding the evidence base to examine factors driving decisions pertaining to coping would enriching our understanding of *why* individuals employ a particular coping approach.

Third, research focused on forcibly displaced youth from Venezuela, Somalia and Central African Republic are currently needed to better align the evidence base with the top refugee countries of origin (as of 2022). As these countries will change over time, monitoring the flows of global displacement will remain important in choosing where research resources are directed.

Despite recognition of mental health as a priority issue for forcibly displaced youth, key knowledge gaps are evident regarding the prevalences of CMDs for this group, their stressors, and coping approaches. Defining the evidence base on these areas is critical to developing interventions to best target stressors, support coping approaches, and enhance mental health for this vulnerable group in LMICs. Thus, findings may be useful for policymakers, mental health professionals, and other stakeholders involved in mental health service development and provision for forcibly displaced youth in LMICs.

This review provides a post-COVID update on stressors, mental health and coping amongst forcibly displaced youth in LMICs. The largest source of stress for this group has been the economic downturn resulting from the pandemic. As such, a specific focus on youth-targeted educational and employment programs should be prioritised by mental health professionals and other stakeholders involved in mental health intervention development. Such a focus addresses the context of reception within the risk and resilience model (Suárez-Orozco et al., 2018), thereby bolstering resilience amongst forcibly displaced youth in LMICs.
